# Effect of clinical symptoms on the indication for selective neck dissection for N0 carcinomas of the parotid gland

**DOI:** 10.3892/ol.2014.2137

**Published:** 2014-05-12

**Authors:** TAKASHI MARUO, YASUSHI FUJIMOTO, KENJI YOSHIDA, MARIKO HIRAMATSU, ATSUSHI SUZUKI, NAOKI NISHIO, MARIKO SHIMONO, TSUTOMU NAKASHIMA

**Affiliations:** Department of Otorhinolaryngology, Nagoya University Graduate School of Medicine, Nagoya, Aichi 466-8550, Japan

**Keywords:** parotid gland cancer, neck dissection, clinical symptoms

## Abstract

Lymph node metastasis is a major prognostic factor in parotid carcinoma, however, the pre-operative diagnosis of occult nodal metastasis is difficult in clinical N0 (cN0) parotid cancer patients. In addition, the indication of neck dissection in T1-3 cN0 patients is controversial. The current study investigated 17 patients with clinical T1-3 cN0 parotid cancer, and analyzed the correlation between patient symptoms/findings and pathological N status/tumor histological grade. In the statistical analysis, pain was found to significantly correlate with neck metastasis. Furthermore, cN0-staged patients without pain exhibited no neck metastasis. However, no significant correlation was identified between patient symptoms or findings and histological grade. These results indicate the possibility that selective neck dissection can be omitted for T1-3 cN0-staged patients without pain.

## Introduction

Carcinoma of the parotid gland represents ~2% of all head and neck cancers (1). Cancer of the parotid gland is classified into several histological types, and the grade of parotid cancer varies with the histological type. As a result, certain tumors are slow growing, while others are more aggressive. Treatment is typically surgery, which may be followed by radiation therapy, while chemotherapy can be effective in treating later stage cancers.

Cervical nodal metastases are a major adverse prognostic factor (2,3). High tumor grade, extraparotid extension, a tumor size of ≥4 cm and facial nerve involvement are associated with nodal disease (4). Even if the option of selective neck dissection is determined on the basis of histological grade or primary tumor stage (T stage), an accurate pre-operative assessment of the histological grade may be difficult in patients with parotid carcinoma (5,6). In previous studies, it has been reported that more than half of T4-staged parotid carcinoma patients exhibit neck node metastasis (6). As a result, we recommend surgery with elective neck dissection (END) for T4-staged parotid carcinoma. However, the treatment of T1–T3-staged patients remains controversial.

The National Comprehensive Cancer Network guidelines (7) show that the characteristics of a benign tumor include a mobile superficial lobe, slow growth and no pain, as well as a lack of or intact neck nodes. This has shown that in addition to neck nodes and facial nerve paralysis, clinical symptoms are also significant. The present study was conducted to determine the correlation between clinical symptoms and nodal metastasis and clinical outcome in patients with T1-3 parotid cancers. The specific point of interest was the investigation of the pretreatment clinical symptoms of regional lymph node stage (N stage), and in particular clinical N0 (cN0)-staged patients at a high risk for occult metastasis who may potentially benefit from selective neck treatment.

## Patients and methods

### Population data

**Figure 1:**
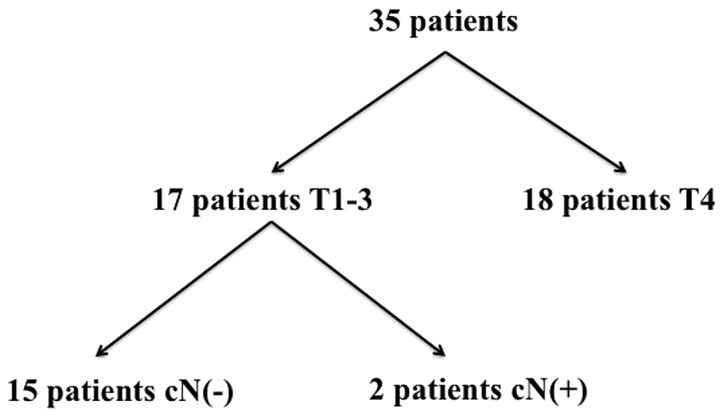
Parotid cancer patient characteristics and pre-operative clinical staging. cN, clinical N stage.

Between 2003 and 2011, 35 previously untreated patients with carcinoma of the parotid gland received definitive treatment at the Nagoya University Hospital (Nagoya, Japan). In the present study, 17 T1-3-staged patients (Fig. 1, Table I) of the 35 patients were analyzed according to the inclusion critearia of T and N stage, including 11 males and six females who ranged in age between 27 and 80 years. The median follow-up duration was 47 months. T and N staging, histological type and four clinical findings (tumor mobility, neck pain, facial palsy and skin invasion) were analyzed. The clinical findings were compared with the presence of lymph node metastasis and histological type grade. Patients provided written informed consent.

### TN staging, histological grades and diagnosis

Seven of the 17 patients (41.2%) were classified as T1, six (35.3%) as T2 and four (23.5%) as T3 (Table I). In addition, 15 patients were regarded as cN0 and two as N2b (Fig. 1). Selective neck dissection was performed for all surgical parotid cancer patients, including stage N0 patients. The histological grade classified patients into three groups: High, intermediate and low grade. Of the 17 patients, two patients were classified as high, eight (47.1%) as intermediate and seven (41.2%) as low grade. Each histological type for all the patients and the grade-dependent classification are shown in Table II.

### Evaluation of clinical symptoms and findings

All the symptoms of the T1-3 N0 patients (n=15) were analyzed, and tumor mobility was analyzed in 11 patients. As the clinical reports of four patients did not contain adequate data, they were excluded from the analysis. In total, five of the 11 patients exhibited poor tumor mobility, and five out of 15 patients exhibited pain. None of the patients exhibited facial palsy or skin invasion (Table III). The correlation between these symptoms, and LN metastasis and histological grade were evaluated.

### Treatment

All patients were initially treated by parotid resection. Neck dissection was performed on all patients, including N0 patients, in which levels I, II and III were resected and examined histologically. Facial nerves without tumor invasion were preserved when possible. Post-surgical irradiation was performed in five patients (29.4%) with a high-grade tumor or positive surgical margin.

### Statistical analysis

The statistical analysis was performed using Pearson’s χ^2^ test, and the survival expectation was calculated by the Kaplan-Meier test. P<0.05 was considered to indicate a statistically significant difference. All statistical analyses were performed using JMP 8.0 software (SAS Institute, Inc., Cary, NC, USA).

## Results

### Pathological staging

According to the histological examination of the neck node at the pretreatment examination, 13 patients were determined as pathological N0 (pN0), 15 patients as cN0 and two patients as pN1 (Table IV).

### Comparison between clinical findings and neck metastases

Three patients presented with pain, but without neck metastases, while two patients presented with neck metastasis. In addition, 10 patients were without pain and neck metastasis. No patients were identified without pain, but with neck metastasis. A significant correlation was identified between pain and neck metastasis (P<0.05; Table V).

Six patients with good tumor mobility were without neck metastasis, while no patients were identified with neck metastasis. Three of the 11 patients exhibited poor mobility of the tumor without neck metastasis, and two patients with poor mobility and neck metastasis. No significant correlation was identified between the mobility of the tumor and neck metastasis (P=0.08; Table V).

### Comparison between clinical findings and histological grade

Two patients with pain had a low histological grade tumor, while three patients had an intermediate grade tumor. In addition, five patients without pain had a low histological grade tumor and five patients had an intermediate grade tumor. No significant correlation was identified between pain symptoms and the histological tumor grade (P=0.14; Table VI).

Furthermore, four patients with good mobility of the tumor had a low histological grade tumor and two patients had an intermediate grade tumor. Two patients presented with poor tumor mobility and a low histological grade tumor, and three with an intermediate grade tumor. No significant correlation was identified between the mobility of the tumor and the histological tumor grade (P=0.37; Table VI).

## Discussion

Previous studies have suggested several prognostic factors for parotid carcinoma. In particular, histological grade and T and N staging have been analyzed in a number of studies (8–12). In the present study, N staging was evaluated as a prognostic factor, and it was considered whether END should be added to the parotid resection. It is commonly accepted that END must be performed in patients with a histologically high-grade malignancy, T3 or higher stage, facial palsy or extraparotid invasion, however, the indication of neck dissection is controversial (5).

The incidence of cervical nodal disease in parotid carcinoma is 14–16% (13). High tumor grade, extraparotid extension, a tumor size of ≥4 cm, pain and facial nerve involvement are associated with nodal disease (13,14).

Stodulski *et al* (15) analyzed the clinical signs and symptoms (facial palsy, skin invasion, neck lymphadenopathy, pain, tumor fixation and rapid tumor growth) as prognostic factors, and as a result, concluded that facial nerve palsy and skin infiltration are significant independent prognostic factors. In the current study, the patient findings and symptoms were considered to be of possible prognostic value for N status. Therefore, pain, tumor mobility, facial palsy and skin invasion of the tumor were analyzed.

In these analyses, only pain exhibited a significant correlation with N status. In previous studies, pain symptoms have shown no significant prognostic value (15). The current study is the first to report that pain symptoms exhibit a significant correlation with patients with or without neck metastasis. In particular, no patients without pain were identified with neck metastasis. This result indicated that T1-3, cN0-staged parotid carcinoma patients without pain may be treated by parotid resection only, without END. Other studies have also reported that pain symptoms can be divided into two types; earache and headache (16,17). The current study did not investigate pain in this manner and therefore, these types of pain must be analyzed in the future.

A number of studies have also reported that histological grade is a significant prognostic factor (1,10,11,18). However, histological types and grades are difficult to evaluate prior to surgery. The histological grades are often classified into three types (high, intermediate and low grade), and have show significant differences in prognosis in previous studies (1,19,20). However, in the present study, no significant difference was identified between pain symptoms and tumor grade. The lack of high-grade tumors in T1-3, N0 patients may have led to this result and thus, follow-up and analysis are required.

The results of the current study indicate that T1-3, cN0-staged patients without pain exhibit no neck metastasis. It may be possible that selective neck dissection can be omitted for T1-3, cN0-staged patients without pain. By contrast, T1-3, cN0-staged patients with pain may exhibit occult neck metastasis and a poor prognosis. In conclusion, pain may be the only prognostic symptom that is useful in the pretreatment diagnosis of parotid carcinoma.

## Figures and Tables

**Table I:** Analysis of patients with parotid cancer.

Patient no.	Gender	Age, years	T stage	N stage
1	Male	72	2	0
2	Male	65	3	0
3	Male	44	1	0
4	Male	62	1	0
5	Female	60	2	0
6	Male	53	2	0
7	Male	59	3	2b
8	Male	80	2	0
9	Male	58	3	2b
10	Female	42	2	0
11	Female	43	2	0
12	Male	65	1	0
13	Male	27	1	0
14	Female	75	1	0
15	Male	64	1	0
16	Female	65	1	0
17	Female	66	3	0

**Table II:** Histological types and grade classified into three groups.

Patient no.	Histological type	Grade
1	Adenoid cystic carcinoma	Intermediate
2	Adenoid cystic carcinoma	Intermediate
3	Adenoid cystic carcinoma	Intermediate
4	Mucoepidermoid carcinoma	Low
5	Carcinoma ex pleomorphic adenoma	Low
6	Mucoepidermoid carcinoma	Low
7	Salivary duct carcinoma	High
8	Epithelial myoepithelial carcinoma	Intermediate
9	Salivary duct carcinoma	High
10	Mucoepidermoid carcinoma	Intermediate
11	Adenoid cystic carcinoma	Intermediate
12	Adenocarcinoma NOS	Low
13	Epithelial myoepithelial carcinoma	Intermediate
14	Mucoepidermoid carcinoma	Low
15	Adenocarcinoma NOS	Low
16	Mucoepidermoid carcinoma	Intermediate
17	Adenoid cystic carcinoma	Low

NOS, not otherwise specified.

**Table III:** Patient findings and symptoms.

Clinical symptoms	n
Pain (n=15)	5
Poor mobility (n=11)	7
Skin invasion (n=15)	0

**Table IV:** N staging following pathological examination of resected lymph node (pN).

	pN status, n				
	
cN status	pN0	pN1	pN2	pN3	Total
cN0	13	2	0	0	15

pN, pathological N stage; cN, clinical N stage.

**Table V:** Symptoms compared with pN status.

A, Pre-operative pain symptoms				

Variable	Neck metastasis (+), n	Neck metastasis (−), n	Total, n	P-value^a^
Pain	2	3	5	<0.05
No pain	0	10	10	
Total	2	13	15	

B, Pre-operative tumor mobility				

Variable	Neck metastasis (+), n	Neck metastasis (−), n	Total, n	P-value^a^

Poor mobility	2	3	5	0.08
Good mobility	0	6	6	
Total	2	9	11	

a Pearson’s χ^2^ test.

pN, pathological N stage.

**Table VI:** Pain and mobility symptoms compared with histological grade.

Grade	Pain, n	No pain, n	Poor mobility, n	Good mobility, n
Low	2	5	2	4
Intermediate	3	5	3	2
High	0	0	0	0
